# Epigenetic mechanisms and stress coping in mood disorders

**DOI:** 10.1192/j.eurpsy.2022.550

**Published:** 2022-09-01

**Authors:** L. Hogea, A.M. Ulucean, L. Nussbaum

**Affiliations:** “Victor Babes” University of Medicine and Pharmacy, Department Of Neurosciences, Timisoara, Romania

**Keywords:** mood disorders, stress, coping, epigenetic

## Abstract

**Introduction:**

Experimental data from both clinical and preclinical studies have unequivocally shown positive correlations between stress and depression, stress, depression and epigenetic changes.

**Objectives:**

The aim of this research is to analyze clinical trials on coping mechanisms and their interaction with epigenetic mechanisms in patients with mood disorders. Generally, we studied the interaction between these two mechanisms and its effects on the onset, recurrence and progression of these disorders.

**Methods:**

109 articles were analyzed, of which 37 were considered relevant. 72 studies were excluded based on titles and abstracts. Regarding the coping mechanisms, 10 longitudinal and cross-sectional studies were selected. Longitudinal studies are defined here by a follow-up period longer than 6 months.

**Results:**

There is a consistent association in the literature between the degree of methylation of the NR3C1 gene, stress and affectivity disorders. The analyzed studies showed that methylation of the NR3C1 gene is associated with both stress and mood disorders. FKBP5 influences glucocorticoid receptor sensitivity and stress response. SLC6A4 gene methylation has been systematically associated with stress and affectivity disorders. Higher BDNF methylation has also been found in people who report high levels of stress at work. The data collected suggest that SKA2 methylation may serve as a biomarker for certain features of depression, such as suicidal ideation, and is not directly involved in the etiology of mood disorders.

**Conclusions:**

The results suggest that environmental stress and adversity in early childhood may change biological systems through epigenetic mechanisms and have long-term consequences, increasing the risk for unfavorable prognosis of mood disorders.

**Disclosure:**

No significant relationships.

